# A Novel PARP Inhibitor YHP-836 For the Treatment of BRCA-Deficiency Cancers

**DOI:** 10.3389/fphar.2022.865085

**Published:** 2022-07-13

**Authors:** Tingting Du, Zhihui Zhang, Jie Zhou, Li Sheng, Haiping Yao, Ming Ji, Bailing Xu, Xiaoguang Chen

**Affiliations:** ^1^ State Key Laboratory of Bioactive Substances and Functions of Natural Medicines, Institute of Materia Medica, Chinese Academy of Medical Sciences and Peking Union Medical College, Beijing, China; ^2^ Beijing Key Laboratory of New Drug Mechanisms and Pharmacological Evaluation Study, Institute of Materia Medica, Chinese Academy of Medical Sciences and Peking Union Medical College, Beijing, China; ^3^ Beijing Key Laboratory of Non-Clinical Drug Metabolism and PK/PD Study, Institute of Materia Medica, Chinese Academy of Medical Sciences and Peking Union Medical College, Beijing, China

**Keywords:** PARP inhibitor, BRCA, chemotherapy agent, PARP (poly(ADP-ribose) polymerase, cancer therapy

## Abstract

PARP inhibitors have clinically demonstrated good antitumor activity in patients with BRCA mutations. Here, we described YHP-836, a novel PARP inhibitor, YHP-836 demonstrated excellent inhibitory activity for both PARP1 and PARP2 enzymes. It also allosterically regulated PARP1 and PARP2 via DNA trapping. YHP-836 showed cytotoxicity in tumor cell lines with BRCA mutations and induced cell cycle arrest in the G2/M phase. YHP-836 also sensitized tumor cells to chemotherapy agents *in vitro*. Oral administration of YHP-836 elicited remarkable antitumor activity either as a single agent or in combination with chemotherapy agents *in vivo*. These results indicated that YHP-836 is a well-defined PARP inhibitor.

## Introduction

The poly-adenosyl-ribose polymerases (PARPs) are a family of enzymes that regulate protein post-translational modification by transferring the ADP-ribose group to target proteins (Gibson and Kraus, 2012; Bai, 2015). PARP1 and PARP2 are the main PARP enzymes involved in base-excision repair of DNA single-strand breaks. PARP1 also plays roles in other DNA damage repair including nucleotide excision repair, nonhomologous end-joining repair, and microhomology-mediated end-joining repair (Couto et al., 2011; Patel et al., 2011). Targeting PARP is an attractive oncologic therapy as genomic instability is a hallmark of cancer that drives tumorigenesis and progression (Hanahan and Weinberg, 2011; Do and Chen, 2013). Indeed, inhibition of PARP1/2 is synthetically lethal with homologous recombination deficiency (HRD) including germline *BRCA1* or *BRCA2* (*gBRCA*) mutations or non-germline HRD-enriched tumors (Bryant et al., 2005; Farmer et al., 2005; Underhill et al., 2011).

PARP inhibitors, including olaparib, rucaparib, niraparib, talazoparib, and pamiparib, have clinically demonstrated significant and sustained antitumor responses as a single agent in patients with *gBRCA* mutation tumors with a favorable toxicity profile (Brown et al., 2016; Spriggs and Longo, 2016; Yuan et al., 2017; Mateo et al., 2019; Markham, 2021; Paluch-Shimon and Cardoso, 2021). PARP inhibitors have also been shown to sensitize tumors cells with chemotherapy drugs such as alkylating agents, topoisomerase I inhibitors, and anti-angiogenesis agents (Plummer et al., 2013; Norris et al., 2014; Ivy et al., 2016; Matulonis and Monk, 2017; Lu et al., 2018; Bizzaro et al., 2021; Chatterjee et al., 2021). Recently, PARP1/2 inhibitors have been reported to be involved in cancer immunity *via* various mechanisms (Lee and Konstantinopoulos, 2019; Lampert et al., 2020; Lee and Konstantinopoulos, 2020). In ovarian cancer, PARP1/2 inhibitors exhibited antitumor immunity *via* a stimulator of interferon genes (STING) in a dependent manner (Ding et al., 2018). PARP1/2 inhibitors yielded encouraging results in combination with immune checkpoint inhibitors by promoting neoantigen release, increasing tumor mutational burden, and enhancing PD-L1 expression (Ding et al., 2019; Lampert et al., 2020). These promising data in preclinical and early clinical studies provide a wide clinical application of PARP1/2 inhibitors in the future.

Here, we reported a novel PARP1/2 inhibitor, YHP-836. YHP-836 showed the inhibitory effect of both enzymes and DNA trapping against PARP1 and PARP2. YHP-836 exhibited cytotoxicity and induced cell cycle arrest in the G2/M phase in BRCA-deficient tumor cells. The antitumor roles of YHP-836 alone or in combination with chemotherapy agents were evaluated *in vitro* and *in vivo*. Oral administration of YHP-836 elicited good antitumor activity *in vivo*.

## Materials and Methods

### Reagents and Antibodies

YHP-836 was synthesized in-house. PARP1/2 inhibitor olaparib was purchased from TargetMol, United States. Temozolomide (TMZ), topotecan, cisplatin, and adriamycin were purchased from J&K Scientific (Beijing, China). Anti-γH2AX and anti-RAD51 were obtained from Cell Signaling Technology (Danvers, MA, United States). An anti-β-actin antibody was purchased from Santa Cruz Biotechnology (Dallas, TX, United States). Anti-PARP1 and anti-PARP2 antibodies were from Abcam (Cambridge, United Kingdom). Anti-PAR antibody and HT PARP pharmacodynamic assay kit were purchased from Trevigen (Gaithersburg, MD, United States). The subcellular protein fractionation kit was purchased from Thermo Scientific (Rockford, IL, United States).

### Cell Culture

The cell lines MCF-7, MDA-MB-436, MDA-MB-231, MDA-MB-453, MDA-MB-468, SUM149PT, Capan-1, and OVCAR8 were obtained from the Cell Resource Centre at the Institute of Medical Sciences, Peking Union Medical College. UWB1.289 and UWB1.289 + BRCA cells were obtained from ATCC. MX-1 was available in our lab. All cell lines were cultured in a humidified atmosphere of 5% CO_2_ at 37 C. MDA-MB-436, MDA-MB-231, MDA-MB-453, MDA-MB-468, and OVCAR8 cells were in RPMI1640 medium (Gibico, TX, United States) with 10% FBS and 1 × penicillin–streptomycin. MCF-7 and MX-1 cells were in Dulbecco’s modified eagle medium (Gibico) with 10% FBS and 1 × penicillin–streptomycin. The SUM149PT cell was cultured in Ham’s F-12 medium containing 5% FBS, 10 μg/ml insulin, 1 × penicillin–streptomycin and supplemented with 0.5 μg/ml hydrocortisone. The Capan-1 cell was in Iscove’s modified Dulbecco’s medium (Gibico) with 10% FBS and 1 × penicillin–streptomycin. According to ATCC handling information, UWB1.289 was cultured in a medium containing 50% RPMI1640 medium and 50% MEGM (MEBM basal medium and SingleQuot additives) (Lonza, Basel, Switzerland) with a final concentration of 3% FBS and 1 × penicillin–streptomycin. UWB1.289 + BRCA1 was in the same medium condition as UWB1.289 with 200 μg/ml G418.

### PARP1/2 Enzymatic Assay

The enzymatic assay of PARP1 and PARP2 was measured as described before (Zhu et al., 2014; Yao et al., 2015). Briefly, 100 μl of histone (10 μg/ml) in assay buffer was coated in a clear flat-bottom 96-well plate at 4°C overnight. After a washing step, 35 μl of NAD^+^ (25 pmol NAD^+^), 10 μl of PARP1 or PARP2 (0.05 unit), and 5 μl of YHP-836 or olaparib (3-fold dilution from 100 nM) were added and incubated at room temperature for 1 h. Then, the PAR product was determined. IC_50_ values of compounds were calculated (Zhu et al., 2014).

### Cell Viability Assay

Cell viability was assessed using 3-(4,5-dimethylthiazol-2-yl)-2,5-diphenyltetrazolium bromide (MTT; Sigma Aldrich, Darmstadt, Germany). Briefly, 2000 cells/well were seeded into a 96-well plate. After incubation overnight, the cells were treated with different concentrations (1.5625, 3.125, 6.25, 12.5, 25, and 50 μM) of YHP-836 or olaparib with three replicates for 72 h. Then, MTT solution was added and incubated for 4 h. Then, MTT solution was gently removed and 100 μl DMSO was added. Absorbance values were measured at the wavelength of 570 nm using a microplate reader (Biotek Instruments, Inc., United States). The half maximal inhibitory concentration (IC_50_) was calculated using GraphPad Prism v8.0.1 (La Jolla, CA). For the combination assay, diluted concentrations of chemotherapy agents were added with 2.5 or 5 μM YHP-836.

### PARP-DNA Trapping Analysis

MX-1 cells were treated with various concentrations of YHP-836 (1, 5, and 10 μM) for 24 h. Then, the cells were harvested. The nuclear soluble and chromatin sections were collected following the protocol of the subcellular protein fractionation kit. Then, the subcellular fractions were tested by immunoblotting.

### Cell Cycle Analysis

Flow cytometry assays were used to analyze the cell cycle distribution as previously reported (Ji et al., 2018). In brief, MX-1 and MCF-7 cells were dispensed into six-well plates at a density of 50,000 cells/well. After growing overnight in a humidified atmosphere of 5% CO_2_ at 37°C, the cells were treated with indicated concentrations of YHP-836 (5 and 10 μM) or olaparib (10 μM) for 24 h. Then, the cells were harvested and fixed with ice cold 70% ethanol overnight at −20°C, washed with PBS, and stained with propidium iodide (PI) solution containing 20 mg/ml PI and 20 mg/ml RNaseA in PBS for 30 min. DNA contents were measured using the BD fluorescence-activated cell sorting (FACS) verse flow cytometer (BD Biosciences, NJ, United States), and the cell cycle distribution was analyzed.

### Immunoblotting Analysis

Cells or mice tumor tissues were collected and lysed in RIPA lysate buffer supplemented with 1% protease inhibitor cocktail and 1% phosphatase inhibitor cocktail (TargetMol, United States). Lysates were then centrifuged at 12,000 g for 30 min. Proteins were quantified using a bicinchoninic acid (BCA) assay kit (Solarbio, Beijing, China). Resultant samples containing equal amounts of proteins were subjected to sodium dodecylsulfate polyacrylamide gel electrophoresis (SDS-PAGE) and transferred to a polyvinylidene fluoride membrane (Millipore, Darmstadt, Germany). The membrane was blocked with TBST buffer containing 5% non-fat milk for 30 min and incubated with appropriate primary antibodies (1:1000 dilution) in TBST at 4°C overnight. After washing with TBST, the membrane was incubated with horseradish peroxidase (HRP)–conjugated secondary antibodies (1:2000 dilution; Cell Signaling Technologies, Boston, MA) for 1 h at room temperature. Bound proteins were visualized using enhanced chemiluminescence and detected using ImageQuant LAS 4000 software.

### Immunofluorescent Staining

Cells at the appropriate density were cultured in the confocal culture dishes and treated with YHP-836 alone or in combination with TMZ for 24 h. The cells were then washed in PBS and fixed with 4% PFA at 4°C for 30 min. The permeabilization was carried out with 0.1% TritonX-100 for 10 min. Anti-RAD51 and anti-γH2AX antibodies at 1:200 dilution were dissolved in 1% bovine serum albumin (BSA). The cells were incubated with primary antibody solutions for 2 h at room temperature. Secondary Alexa Fluor 594 or 488 antibodies were used to bind and visualize the primary antibody. The culture dishes were then mounted using Origene ZLI-9556 mounting medium with DAPI. The photographs were taken using the Olympus FV1000MPE Confocal microscope.

### Animal Study

Female Balb/c athymic nude mice (8–10 weeks old) were subcutaneously implanted with 1 × 10^7^ MDA-MB-436, MX-1, or MCF-7 cells in 0.1 ml matrigel solution in the right flank of nude mice. After 2 weeks, the tumor tissue was harvested aseptically, and tumor cells were extracted from tissue homogenate. Then, the mice were implanted with 5 × 10^6^ tumor cells each. Seven days later, when the average tumor volumes reached 100–300 mm^3^, the mice were randomized and received treatment (Day 0). For the MDA-MB-436 xenograft model, mice were orally administered vehicle or YHP-836 at a dose of 50, 100, or 150 mg/kg dissolved in 0.5% CMC twice daily for 25 days. In the MX-1 xenograft model, mice were orally administered vehicle (once per day for 5 days), TMZ at the dose of 50 mg/kg/day (once per day for 5 days), or YHP-836 at the dose of 25 mg/kg/day alone (once per day for 5 days) or in combination with TMZ (once per day for 5 days). For other chemotherapy agents, CDDP at a dose of 6 mg/kg was intraperitoneally injected once per week and ADM at a dose of 5 mg/kg was intraperitoneally administrated every 3 days. YHP-836 was orally administered once per day alone or combined with chemotherapy agents for 9 days. In the MCF-7 xenograft model, YHP-836 at the dose of 25 mg/kg/day was administered alone (once per day daily for 24 days) or combined with TMZ (once per day daily for 5 days). Tumor volumes and body weights were monitored twice a week. Tumor volume was calculated as V = 1/2 × L × W^2^, where L is the maximum length of the tumor, and W is the maximum width of the tumor. The mice were euthanized, and the tumor tissues were collected for immunoblotting or ELISA assay.

All procedures were approved by the Ethics Committee for Animal Experiments of the Institute of Materia Medica, Chinese Academy of Medical Sciences & Peking Union Medical College, and conducted following the Guidelines for Animal Experiments of Peking Union Medical College.

### Statistical Analysis

Most statistical analyses were performed utilizing GraphPad Prism 8.0.1 (La Jolla, CA), and significance levels were evaluated using analysis of variance (ANOVA) or t-tests, as appropriate. Here, we distinguish between three *p* values of significance (^***^ *p* < 0.001, ^**^ *p* < 0.01, and ^*^ *p* < 0.05, respectively).

## Results

### YHP-836 Inhibited PARP1/2 Activity *In Vitro*

**FIGURE 1:**
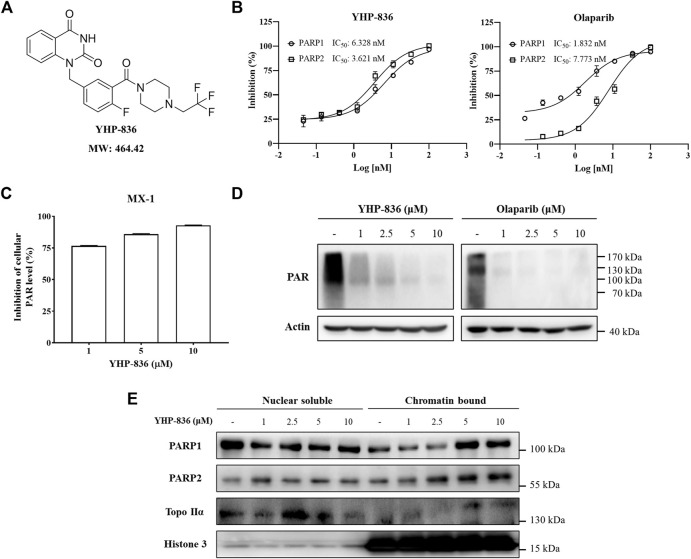
The structure of YHP-836 and its activity. **(A)** The chemical structure of YHP-836. **(B)** The inhibitory effect of YHP-836 and olaparib on PARP1 and PARP2. **(C)** The activity of YHP-836 against intracellular PAR levels in MX-1 cells. MX-1 cells were treated with indicated concentrations of YHP-836 for 12 h. **(D)** The inhibitory effect of YHP-836 and olaparib on PAR levels in MX-1 cells by immunoblotting. MX-1 cells were exposed to indicated concentrations of YHP-836 or olaparib for 24 h. **(E)** PARP DNA-trapping ability of YHP-836. MX-1 cells were incubated with indicated concentrations of YHP-836 for 24 h.

YHP-836 (Figure 1A) is a novel PARP inhibitor with PARP1 and PARP2 enzymatic IC_50_ values of 6.328 and 3.621 nmol/L, respectively (Figure 1B). The IC_50_ values of olaparib for PARP1 and PARP2 were 1.832 and 7.773 nmol/L. The ELISA assay showed that YHP-836 dose-dependently reduced intracellular PAR levels in MX-1 breast cancer cells (Figure 1C), which reflected the catalytic activity of PARP1 and PARP2. Immunoblotting results also showed the PAR levels were decreased in MX-1 exposed to various concentrations of YHP-836 for 24 h (Figure 1D). Exposed to YHP-836 at the concentration of 1 μM, intracellular PAR levels in cells were notably reduced. PARP inhibitor olaparib was used as a positive control. It was reported that PARP inhibitors can not only inhibit PARP1 and PARP2 catalytic domain, but also allosterically regulate them to bind to damaged single-strand DNA continually and impede the recruitment of DNA damage–related proteins. Thus, we tested the function of YHP-836 on the PARP-DNA complex *via* DNA trapping in MX-1 cells. As shown in Figure 1E, PARP1 accumulated in a dose-dependent manner in the chromatin section after YHP-836 treatment. At the concentration of 5 μM, YHP-836 strongly increased the level of PARP1 binding to chromatin. Similar results were also observed for PARP2. These results indicated that YHP-836 is a definitive PARP1/2 inhibitor.

### The Cytotoxicity of YHP-836 *In Vitro*

**FIGURE 2:**
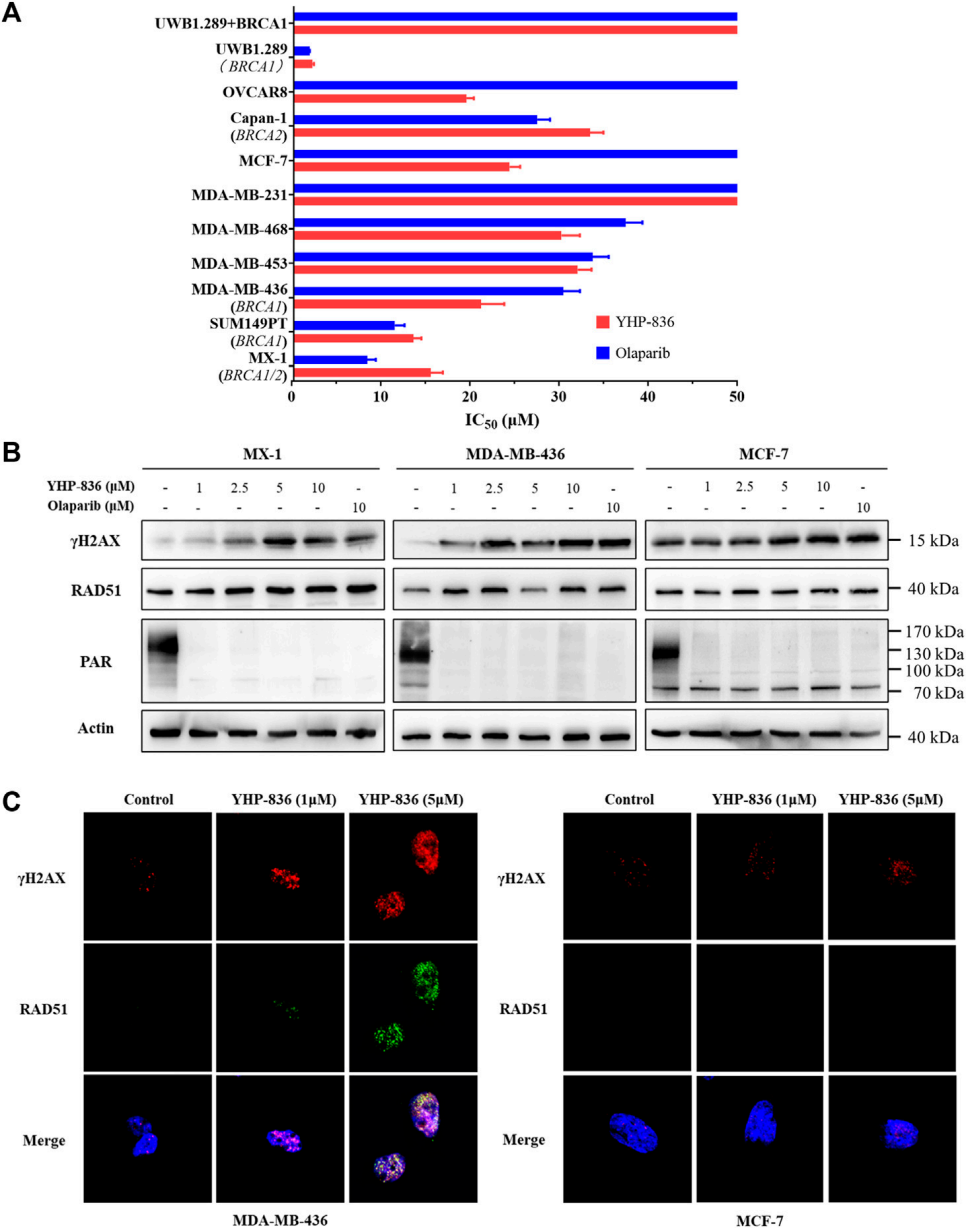
The cytotoxicity of YHP-836. **(A)** The cytotoxic activity of YHP-836 in tumor cell lines. IC_50_ values were indicated as mean ± SD. **(B)** YHP-836 increased the DNA damage marker in breast cancer cells. The cells were exposed to YHP-836 (1, 2.5, 5, and 10 μM) or olaparib (10 μM) for 24 h. **(C)** Immunofluorescent staining of γH2AX and RAD51 in breast cancer cells. Exposure to indicated concentrations of YHP-836 (1 and 5 μM) for 24 h.

As YHP-836 showed enzymatic inhibitory activity against PARP1 and PARP2, we investigated if YHP-836 could suppress cancer cell proliferation with BRCA mutations via synthetic lethality. Cell cytotoxicity was detected in a panel of cancer cells exposed to YHP-836 or olaparib at 72 h. As shown in Figure 2A, YHP-836 inhibited cancer cell growth with BRCA1/2 mutation more effectively than those with wild type. YHP-836 in UWB1.289, a BRCA1-null human ovarian cancer cell, was much more sensitive than this cell-restored wildtype BRCA1 (UWB1.289 + BRCA1). Consistently, in MX-1 cells with the *BRCA1* mutation and *BRCA2* null and in MDA-MB-436 with the *BRCA1* mutation, YHP-836 downregulated the levels of PAR, which was catalyzed by PARP1 and PARP2, consequently increasing the levels of γH2A, a DNA damage marker, and RAD51 protein, which is essential for homologous recombination (Figure 2B). In MCF-7 cells without BRCA mutation, YHP-836 could downregulate the PAR level with increased γH2A, but could not elevate the level of RAD51. Immunofluorescence results indicated that γH2A staining increased in MDA-MB-436 with BRCA1 mutation exposure to YHP-436 at concentrations of 1 and 5 μM (Figure 2C). RAD51 foci also accumulated after treatment. Consistent with immunoblotting results, RAD51 foci did not significantly increase after treatment in MCF-7 cells. Taken together, YHP-836 showed cytotoxicity in cancer cells with BRCA mutations.

### YHP-836 Induced Cell Cycle Arrest

**FIGURE 3:**
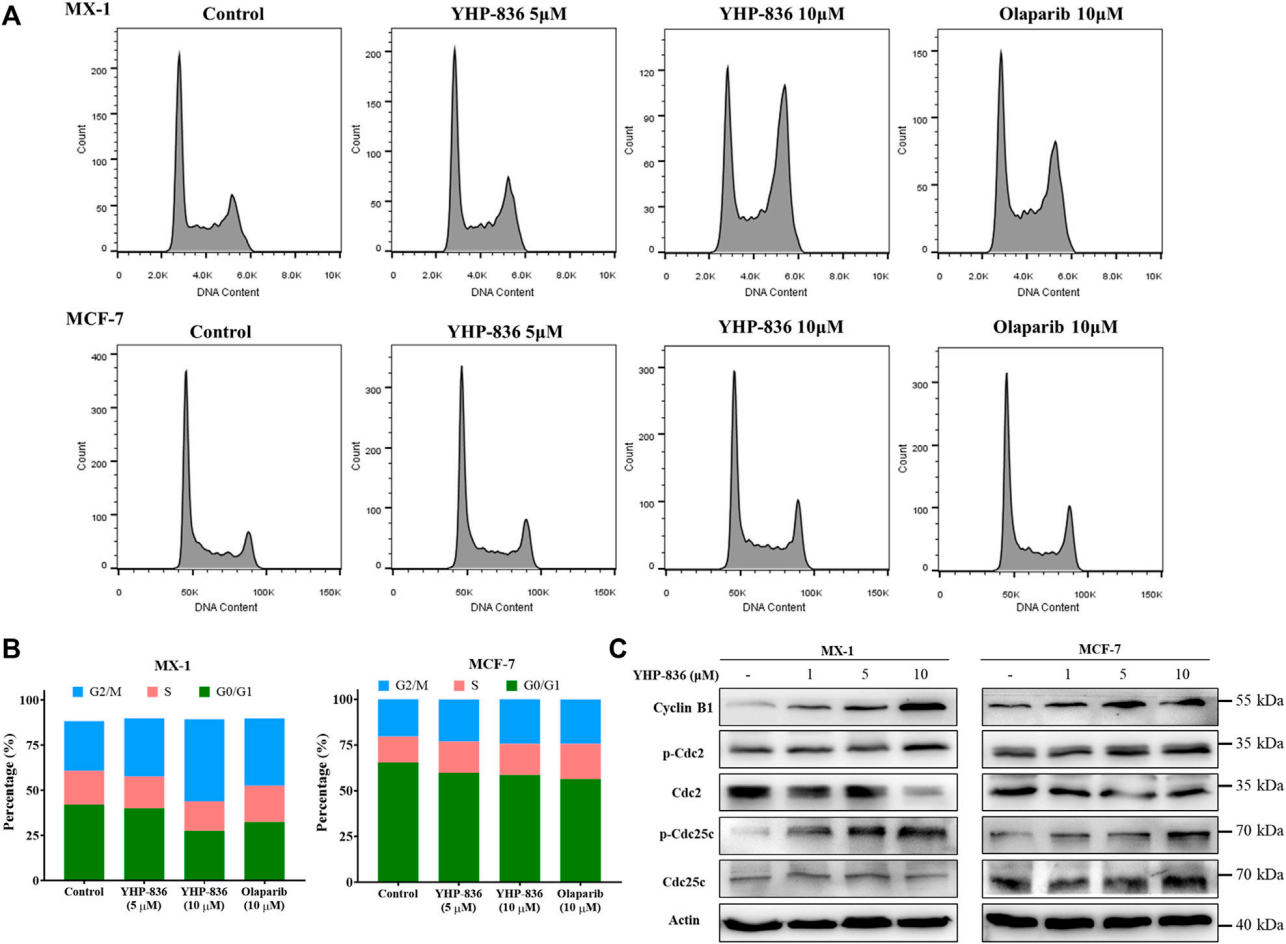
YHP-836 induced cell cycle arrest. **(A,B)** YHP-836 induced cell cycle arrest in MX-1 and MCF-7 in the G2/M phase. Exposure to YHP-836 at the indicated concentrations (5 and 10 μM) or olaparib (10 μM) for 24 h. **(C)** YHP-836 changed the markers of the G2/M cell cycle. The phosphorylation levels of Cdc2, Cdc25c, and protein level of Cyclin B1 were detected via immunoblotting in both MX-1 and MCF-7 cells exposed to YHP-836 for 24 h.

Moreover, cell cycle analysis was performed to evaluate the function of YHP-836 in tumor cells. Both MX-1 and MCF-7 cells were treated with YHP-836 or olaparib. In MX-1 cells, YHP-836 at the concentrations of 5 and 10 μM induced cell cycle arrest in the G2/M phase (Figures 3A,B and Supplementary Figure S1). Olaparib had a similar result. The cell cycle was slightly arrested in MCF-7 cells exposed to YHP-836 or olaparib at the same concentrations. Immunoblotting results showed that cyclin B1 and phosphorylation levels of Cdc2 and Cdc25c dose-dependently increased after YHP-836 treatment in both MX-1 cells and MCF-7 cells (Figure 3C). The levels of Cdc2 dramatically reduced in MX-1 cells exposed to YHP-836 at the concentration of 10 μM. These data indicated that YHP-836 induced cell cycle arrest at the G2/M phase.

### YHP-836 Enhanced Chemotherapy Reagents Cytotoxicity *In Vitro*

**FIGURE 4:**
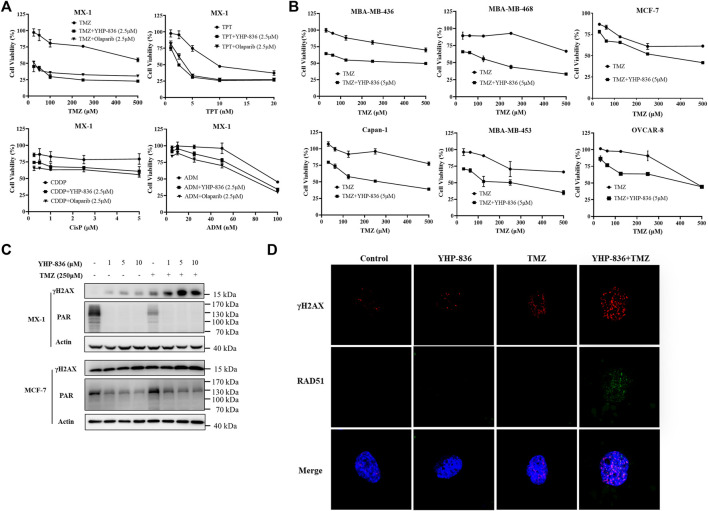
The potential effects of YHP-836 on chemotherapy agents *in vitro*. **(A)** YHP-836 enhanced the cytotoxicity of TMZ, TPT, CDDP, and ADM in MX-1 cells. The cells exposed to TMZ, TPT, CDDP, or ADM alone or in combination with YHP-836 (2.5 μM) or olaparib (2.5 μM) for 72 h. **(B)** YHP-836 enhanced the cytotoxicity of TMZ in various cancer cells. The cells were exposed to TMZ alone or in combination with YHP-836 (5 μM) for 72 h. **(C)** The levels of γH2AX increased in both MX-1 and MCF-7 cells treated with TMZ (250 μM) combined with different concentrations of YHP-836 (1, 5, 10 μM) for 72 h. **(D)** Immunofluorescent staining of γH2AX and RAD51 in MCF-7 cells. The cells were treated with YHP-836 (5 μM), TMZ (250 μM), or a combination for 24 h.

It is reported that PARP inhibitors can potentiate the antitumor effect of chemotherapy agents such as temozolomide (TMZ) and topotecan (TPT). Thus, we detected the combination effect of YHP-836 with chemotherapy agents in MX-1 cells. As shown in Figure 4A and Supplementary Table S1, YHP-836 at the concentration of 2.5 μM enhanced the cytotoxicity of chemotherapy agents including TMZ, TPT, cisplatin (CDDP) and adriamycin (ADM) in MX-1 cells. The synergistic effects were similar to those of olaparib. We also explored these effects on other cells with or without BRCA mutation. At the concentration of 5 μM, YHP-836 exhibited good potential effects with TMZ on serial tumor cells (Figure 4B and Supplementary Table S2). Sequentially, we detected γH2AX levels combined with TMZ in MX-1 cells and MCF-7 cells. YHP-836 also increased the levels of γH2AX together with TMZ (Figure 4C), indicating the enhanced cytotoxicity of TMZ. Confocal analysis also demonstrated that DNA damage loci were accumulated in the nucleus as γH2AX and RAD51 were increased in the combination treatment (Figure 4D and Supplementary Figure S2).

### Antitumor Activity of YHP-836 *In Vivo*

**FIGURE 5:**
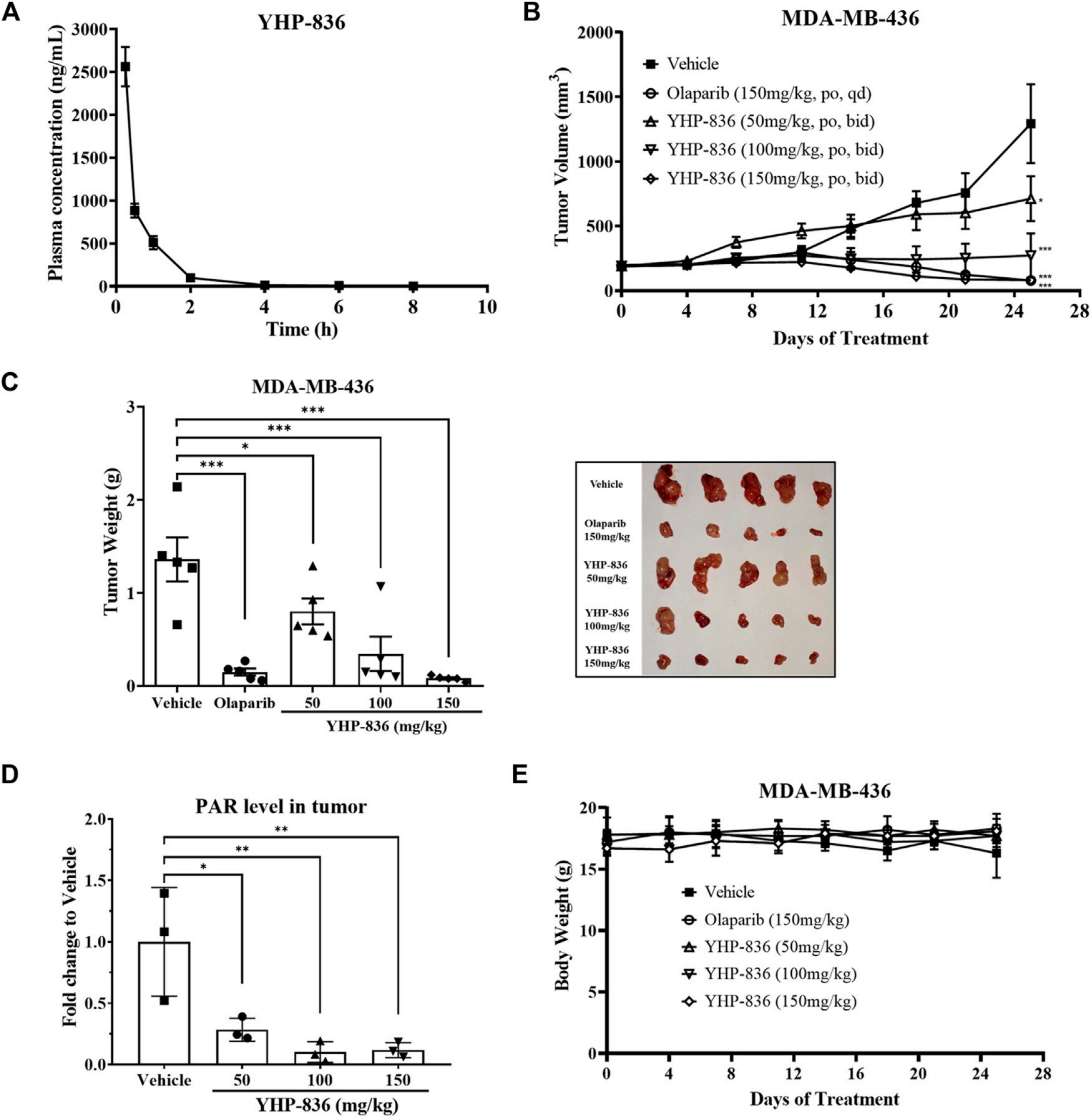
YHP-836 repressed tumor growth in an MDA-MB-436 xenograft model. **(A)** The plasma concentrations of YHP-836 in mice. The mice were orally administered YHP-836 at the dose of 25 mg/kg. Data are presented as mean ± standard deviation (SD), *n* = 3. **(B)** Antitumor activity of YHP-836 in an MDA-MB-436 subcutaneous xenograft model. ANOVA analysis, ^*^ *p* < 0.05, ^***^ *p* < 0.001, compared with the vehicle group. Data are presented as mean ± standard error of the mean (SEM), *n* = 5. **(C)** Tumor weight in an MDA-MB-436 xenograft model. ANOVA analysis, ^*^ *p* < 0.05, ^***^ *p* < 0.001, compared with the vehicle group. Data are presented as mean ± SEM. **(D)** YHP-836 reduced PAR levels in tumor tissues. Data are presented as mean ± SD, *n* = 3. ANOVA analysis, ^*^ *p* < 0.05, ^**^ *p* < 0.01, compared with vehicle group. **(E)** The body weight of mice in an MDA-MB-436 subcutaneous xenograft model. Data are presented as mean ± SD, n = 5.

To confirm the antitumor activity of YHP-836 *in vivo*, we first characterized the pharmacokinetic (PK) properties in mice. As shown in Figure 5A, the maximum plasma concentration of YHP-836 reached about 2500 ng/ml after single oral administration at the dose of 25 mg/kg. However, it was rapidly eliminated, and the shelf-life was not long in mice. Based on the PK properties in mice, we used the MDA-MB-436 xenograft mice model to assess its antitumor activity and the mice were orally administered YHP-836 twice per day or olaparib once per day. As shown in Figures 5B,C, YHP-836 significantly repressed tumor growth in a dose-dependent manner with tumor growth inhibition (TGI) of 41.0, 74.6, and 94.0% for 50 mg/kg, 100 mg/kg, and 150 mg/kg, respectively. Olaparib at the dose of 150 mg/kg once daily also exhibited good antitumor activity with 89.0% TGI. The tumor samples were collected to test the PAR level using ELISA assay. It was observed that YHP-836 dramatically inhibited the PAR synthesis in tumor tissues compared with the vehicle group (Figure 5D). During the experiment, YHP-836 did not cause significant reduction in body weight (Figure 5E).

**FIGURE 6:**
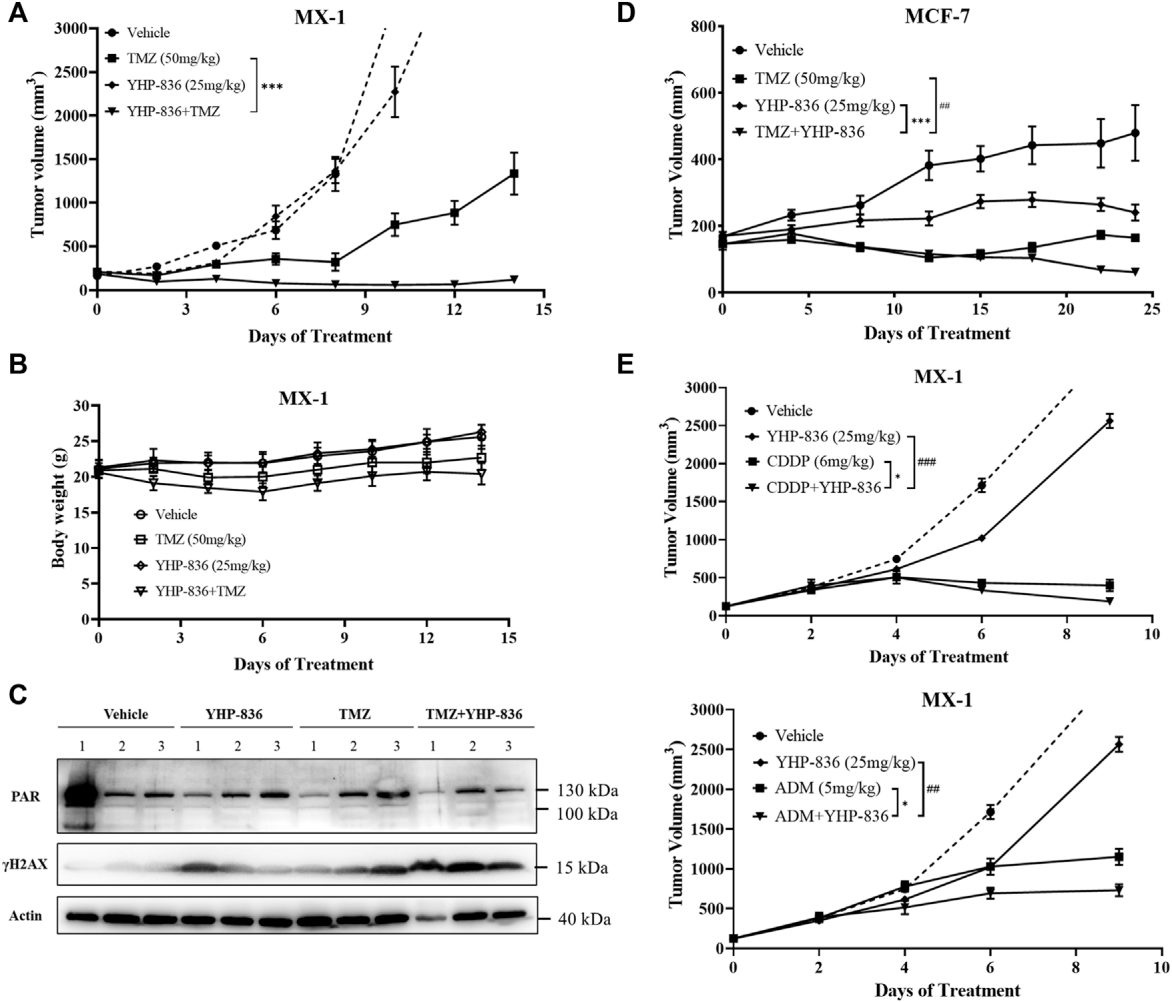
YHP-836 enhanced the antitumor activity of chemotherapy agents in the breast cancer xenograft model. **(A)** Antitumor activity of the combination of YHP-836 and TMZ in an MX-1 subcutaneous xenograft model. *t*-test, ^***^ *p* < 0.01, compared with the TMZ group. Data are presented as mean ± SD, *n* = 7. **(B)** The body weight of mice in an MX-1 subcutaneous xenograft model. Data are presented as mean ± SD, *n* = 7. **(C)** Levels of PAR and γH2AX in tumor tissues from the MX-1 xenograft mice model. Numbers represent three independent xenograft tumors in each group. **(D)** Antitumor activity of the combination of YHP-836 and TMZ in an MCF-7 subcutaneous xenograft model. ANOVA analysis, ^***^ *p* < 0.01, combination group vs. YHP-836 group; ^##^ *p* < 0.01, combination group vs. TMZ group. Data are presented as mean ± SEM, *n* = 5. **(E)** Antitumor activity of the combination of YHP-836 and CDDP or ADM in an MX-1 subcutaneous xenograft model. ANOVA analysis, ^*^ *p* < 0.05, combination group vs. CDDP or ADM group; ^##^ *p* < 0.01, ^###^ *p* < 0.001, combination group vs. YHP-836 group. Data are presented as mean ± SEM, *n* = 5.

As YHP-836 could enhance chemotherapy reagents’ cytotoxicity *in vitro*, we explored the combined antitumor activity of YHP-836 with TMZ *in vivo* as well. YHP-836 at the dose of 25 mg/kg once daily and TMZ at the dose of 50 mg/kg once daily or in combination were orally administered for 5 consecutive days, and the mice were under continuous observation. As shown in Figure 6A, the antitumor activity in the combination group was significantly better than that in the TMZ or YHP-836 group. This effect lasted until the end of the experiment. During the experiment, the body weight in the combination group decreased from day 1 to day 6 and recovered after compound withdrawal (Figure 6B). The tumors were collected for immunoblotting. The results showed that the levels of γH2AX increased in the combination group compared to the TMZ or YHP-836 group (Figure 6C). Next, we investigated the antitumor activity of YHP-836 in combination with MCF-7 mice xenograft models. As shown in Figure 6D, TMZ combined with YHP-836 showed better antitumor activity compared with the groups that received either TMZ or YHP-836 alone. The combination effects of YHP-836 with other chemotherapy agents including cisplatin (CDDP) or adriamycin (ADM) were evaluated in MX-1 mice xenograft models as well. As expected, YHP-836 also enhanced the antitumor activities of these chemotherapy agents (Figure 6E).

## Discussion

PARPs are attractive targets for cancer therapy. PARP inhibitors such as olaparib and pamiparib have been demonstrated to be indicative of monotherapy in patients with ovarian tumor harboring BRCA1 or BRCA2 mutations (Ledermann, 2016; Markham, 2021). The indicators also extended to breast cancers, prostate cancers, and pancreatic cancers with HR deficiency (Kamel et al., 2018; Litton et al., 2018; Charkes, 2019; Aschenbrenner, 2020; de Bono et al., 2020; de Bono et al., 2021). In this report, we presented a novel PARP inhibitor, YHP-836. The compound exhibited good cytotoxicity in cells harboring BRCA mutations. Oral administration of YHP-836 demonstrated remarkable antitumor activity in the MDA-MD-436 breast cancer xenograft model.

Enzymatic inhibition and DNA trapping are important parameters to evaluate the activity of PARP inhibitors. YHP-836 exhibited strong enzymatic inhibitory activity against PARP1 and PARP2, and dose-dependently suppressed the PAR levels in MX-1 cells. YHP-836 also strongly induced DNA trapping in MX-1 cells. Additionally, YHP-836 increased the DNA damage markers γH2A and RAD51 foci *in vitro*. These data characterize YHP-836 as a defined PARP inhibitor. In the MDA-MB-436 mice xenograft model with the BRCA1 mutation, YHP-836 indeed exhibited good antitumor activity by synthetic lethality.

In addition to monotherapy for cancers with HRD, PARP inhibitors are under clinical assessment in combination with other antitumor agents referred for chemotherapy, targeted therapy, and immunotherapy (Plummer et al., 2013; Norris et al., 2014; Matulonis and Monk, 2017; Tomao et al., 2017; Friedlander et al., 2019; Lee and Konstantinopoulos, 2019; Lampert et al., 2020; Palaia et al., 2020; Bizzaro et al., 2021; Waddington et al., 2021). Similar to other PARP inhibitors, YHP-836 also potentiates chemotherapy agents against various tumor cells. In MX-1 and MCF-7 breast cancer xenograft models, YHP-836 could enhance the antitumor activity of TMZ, CDDP, and ADM.

There are several limitations to using YHP-836. First, the selectivity of YHP-836 for PARP1 and PARP2 is not satisfactory. Although clinical benefits of PARP inhibitors have been proved, safety issues such as hematological toxicity need to be addressed (Farres et al., 2013; LaFargue et al., 2019). The next generation of PARP inhibitors is under development, targeting selective PARP1, to remedy the adverse events caused by inhibition of PARP2 (Curtin and Szabo, 2020; Dias et al., 2021; Johannes et al., 2021; Ngoi et al., 2021). Secondly, the PK characteristics of YHP-836 did not support its further development. The shelf-life is very short and maximum plasma concentration is not high, leading to poor bioavailability. Thus, the compound should be further modified.

In conclusion, we reported a novel PARP inhibitor YHP-836 with acceptable antitumor activity *in vitro* and *in vivo*.

## Data Availability

The original contributions presented in the study are included in the article/Supplementary Material; further inquiries can be directed to the corresponding authors.

## References

[B1] Aschenbrenner D. S. (2020). Olaparib Approved for Metastatic Pancreatic Cancer. Am. J. Nurs. 120 (4), 22–23. 10.1097/01.NAJ.0000660008.32418.6c 32218041

[B2] Bai P. (2015). Biology of Poly(ADP-Ribose) Polymerases: The Factotums of Cell Maintenance. Mol. Cell 58 (6), 947–958. 10.1016/j.molcel.2015.01.034 26091343

[B3] Bizzaro F. Fuso Nerini I. Taylor M. A. Anastasia A. Russo M. Damia G. (2021). VEGF Pathway Inhibition Potentiates PARP Inhibitor Efficacy in Ovarian Cancer Independent of BRCA Status. J. Hematol. Oncol. 14, 186. 10.1186/s13045-021-01196-x 34742344 PMC8572452

[B4] Brown J. S. Kaye S. B. Yap T. A. (2016). PARP Inhibitors: the Race Is on. Br. J. Cancer 114 (7), 713–715. 10.1038/bjc.2016.67 27022824 PMC4984871

[B5] Bryant H. E. Schultz N. Thomas H. D. Parker K. M. Flower D. Lopez E. (2005). Specific Killing of BRCA2-Deficient Tumours with Inhibitors of poly(ADP-Ribose) Polymerase. Nature 434, 913–917. 10.1038/nature03443 15829966

[B6] Charkes N. D. (2019). Maintenance Olaparib for Metastatic Pancreatic Cancer. N. Engl. J. Med. 381, 1491. 10.1056/NEJMc1911185 31597027

[B7] Chatterjee S. Sinha S. Molla S. Hembram K. C. Kundu C. N. (2021). PARP Inhibitor Veliparib (ABT-888) Enhances the Anti-angiogenic Potentiality of Curcumin through Deregulation of NECTIN-4 in Oral Cancer: Role of Nitric Oxide (NO). Cell Signal 80, 109902. 10.1016/j.cellsig.2020.109902 33373686

[B8] Couto C. A. Wang H. Y. Green J. C. Kiely R. Siddaway R. Borer C. (2011). PARP Regulates Nonhomologous End Joining through Retention of Ku at Double-Strand Breaks. J. Cell Biol. 194, 367–375. 10.1083/jcb.201012132 21807880 PMC3153639

[B9] Curtin N. J. Szabo C. (2020). Poly(ADP-ribose) Polymerase Inhibition: Past, Present and Future. Nat. Rev. Drug Discov. 19, 711–736. 10.1038/s41573-020-0076-6 32884152

[B10] De Bono J. Mateo J. Fizazi K. Saad F. Shore N. Sandhu S. (2020). Olaparib for Metastatic Castration-Resistant Prostate Cancer. N. Engl. J. Med. 382, 2091–2102. 10.1056/NEJMoa1911440 32343890

[B11] De Bono J. S. Mehra N. Scagliotti G. V. Castro E. Dorff T. Stirling A. (2021). Talazoparib Monotherapy in Metastatic Castration-Resistant Prostate Cancer with DNA Repair Alterations (TALAPRO-1): an Open-Label, Phase 2 Trial. Lancet Oncol. 22, 1250–1264. 10.1016/S1470-2045(21)00376-4 34388386

[B12] Dias M. P. Moser S. C. Ganesan S. Jonkers J. (2021). Understanding and Overcoming Resistance to PARP Inhibitors in Cancer Therapy. Nat. Rev. Clin. Oncol. 18, 773–791. 10.1038/s41571-021-00532-x 34285417

[B13] Ding L. Chen X. Xu X. Qian Y. Liang G. Yao F. (2019). PARP1 Suppresses the Transcription of PD-L1 by Poly(ADP-Ribosyl)ating STAT3. Cancer Immunol. Res. 7, 136–149. 10.1158/2326-6066.CIR-18-0071 30401677

[B14] Ding L. Kim H. J. Wang Q. Kearns M. Jiang T. Ohlson C. E. (2018). PARP Inhibition Elicits STING-dependent Antitumor Immunity in Brca1-Deficient Ovarian Cancer. Cell Rep. 25, 2972–e5. e2975. 10.1016/j.celrep.2018.11.054 30540933 PMC6366450

[B15] Do K. Chen A. P. (2013). Molecular Pathways: Targeting PARP in Cancer Treatment. Clin. Cancer Res. 19, 977–984. 10.1158/1078-0432.CCR-12-0163 23269547 PMC3600578

[B16] Farmer H. Mccabe N. Lord C. J. Tutt A. N. Johnson D. A. Richardson T. B. (2005). Targeting the DNA Repair Defect in BRCA Mutant Cells as a Therapeutic Strategy. Nature 434, 917–921. 10.1038/nature03445 15829967

[B17] Farrés J. Martín-Caballero J. Martínez C. Lozano J. J. Llacuna L. Ampurdanés C. (2013). Parp-2 Is Required to Maintain Hematopoiesis Following Sublethal γ-irradiation in Mice. Blood 122, 44–54. 10.1182/blood-2012-12-472845 23678004 PMC4918799

[B18] Friedlander M. Meniawy T. Markman B. Mileshkin L. Harnett P. Millward M. (2019). Pamiparib in Combination with Tislelizumab in Patients with Advanced Solid Tumours: Results from the Dose-Escalation Stage of a Multicentre, Open-Label, Phase 1a/b Trial. Lancet Oncol. 20, 1306–1315. 10.1016/S1470-2045(19)30396-1 31378459

[B19] Gibson B. A. Kraus W. L. (2012). New Insights into the Molecular and Cellular Functions of poly(ADP-Ribose) and PARPs. Nat. Rev. Mol. Cell Biol. 13, 411–424. 10.1038/nrm3376 22713970

[B20] Hanahan D. Weinberg R. A. (2011). Hallmarks of Cancer: the Next Generation. Cell 144, 646–674. 10.1016/j.cell.2011.02.013 21376230

[B21] Ivy S. P. Liu J. F. Lee J. M. Matulonis U. A. Kohn E. C. (2016). Cediranib, a Pan-VEGFR Inhibitor, and Olaparib, a PARP Inhibitor, in Combination Therapy for High Grade Serous Ovarian Cancer. Expert Opin. Investig. Drugs 25, 597–611. 10.1517/13543784.2016.1156857 26899229

[B22] Johannes J. W. Balazs A. Barratt D. Bista M. Chuba M. D. Cosulich S. (2021). Discovery of 5-{4-[(7-Ethyl-6-Oxo-5,6-Dihydro-1,5-Naphthyridin-3-Yl)methyl]piperazin-1-Yl}-N-Methylpyridine-2-Carboxamide (AZD5305): A PARP1-DNA Trapper with High Selectivity for PARP1 over PARP2 and Other PARPs. J. Med. Chem. 64, 14498–14512. 10.1021/acs.jmedchem.1c01012 34570508

[B23] Kamel D. Gray C. Walia J. S. Kumar V. (2018). PARP Inhibitor Drugs in the Treatment of Breast, Ovarian, Prostate and Pancreatic Cancers: An Update of Clinical Trials. Curr. Drug Targets 19, 21–37. 10.2174/1389450118666170711151518 28699513

[B24] Lafargue C. J. Dal Molin G. Z. Sood A. K. Coleman R. L. (2019). Exploring and Comparing Adverse Events between PARP Inhibitors. Lancet Oncol. 20, e15–e28. 10.1016/S1470-2045(18)30786-1 30614472 PMC7292736

[B25] Lampert E. J. Zimmer A. Padget M. Cimino-Mathews A. Nair J. R. Liu Y. (2020). Combination of PARP Inhibitor Olaparib, and PD-L1 Inhibitor Durvalumab, in Recurrent Ovarian Cancer: a Proof-Of-Concept Phase II Study. Clin. Cancer Res. 26, 4268–4279. 10.1158/1078-0432.CCR-20-0056 32398324 PMC7442720

[B26] Ledermann J. A. (2016). PARP Inhibitors in Ovarian Cancer. Ann. Oncol. 27 (Suppl. 1), i40–i44. 10.1093/annonc/mdw094 27141070

[B27] Lee E. K. Konstantinopoulos P. A. (2019). Combined PARP and Immune Checkpoint Inhibition in Ovarian Cancer. Trends Cancer 5, 524–528. 10.1016/j.trecan.2019.06.004 31474356

[B28] Lee E. K. Konstantinopoulos P. A. (2020). PARP Inhibition and Immune Modulation: Scientific Rationale and Perspectives for the Treatment of Gynecologic Cancers. Ther. Adv. Med. Oncol. 12, 1758835920944116. 10.1177/1758835920944116 32782491 PMC7383615

[B29] Litton J. K. Rugo H. S. Ettl J. Hurvitz S. A. Gonçalves A. Lee K. H. (2018). Talazoparib in Patients with Advanced Breast Cancer and a Germline BRCA Mutation. N. Engl. J. Med. 379, 753–763. 10.1056/NEJMoa1802905 30110579 PMC10600918

[B30] Lu Y. Liu Y. Pang Y. Pacak K. Yang C. (2018). Double-barreled Gun: Combination of PARP Inhibitor with Conventional Chemotherapy. Pharmacol. Ther. 188, 168–175. 10.1016/j.pharmthera.2018.03.006 29621593 PMC6067963

[B31] Markham A. (2021). Pamiparib: First Approval. Drugs 81, 1343–1348. 10.1007/s40265-021-01552-8 34287805

[B32] Mateo J. Lord C. J. Serra V. Tutt A. Balmaña J. Castroviejo-Bermejo M. (2019). A Decade of Clinical Development of PARP Inhibitors in Perspective. Ann. Oncol. 30, 1437–1447. 10.1093/annonc/mdz192 31218365 PMC6771225

[B33] Matulonis U. A. Monk B. J. (2017). PARP Inhibitor and Chemotherapy Combination Trials for the Treatment of Advanced Malignancies: Does a Development Pathway Forward Exist? Ann. Oncol. 28, 443–447. 10.1093/annonc/mdw697 28057663

[B34] Ngoi N. Y. L. Leo E. O'connor M. J. Yap T. A. (2021). Development of Next-Generation Poly(ADP-Ribose) Polymerase 1-Selective Inhibitors. Cancer J. 27, 521–528. 10.1097/PPO.0000000000000556 34904816

[B35] Norris R. E. Adamson P. C. Nguyen V. T. Fox E. (2014). Preclinical Evaluation of the PARP Inhibitor, Olaparib, in Combination with Cytotoxic Chemotherapy in Pediatric Solid Tumors. Pediatr. Blood Cancer 61, 145–150. 10.1002/pbc.24697 24038812 PMC3849815

[B36] Palaia I. Tomao F. Sassu C. M. Musacchio L. Benedetti Panici P. (2020). Immunotherapy for Ovarian Cancer: Recent Advances and Combination Therapeutic Approaches. Onco Targets Ther. 13, 6109–6129. 10.2147/OTT.S205950 32617007 PMC7326187

[B37] Paluch-Shimon S. Cardoso F. (2021). PARP Inhibitors Coming of Age. Nat. Rev. Clin. Oncol. 18, 69–70. 10.1038/s41571-020-00452-2 33239729

[B38] Patel A. G. Sarkaria J. N. Kaufmann S. H. (2011). Nonhomologous End Joining Drives poly(ADP-Ribose) Polymerase (PARP) Inhibitor Lethality in Homologous Recombination-Deficient Cells. Proc. Natl. Acad. Sci. U. S. A. 108, 3406–3411. 10.1073/pnas.1013715108 21300883 PMC3044391

[B39] Plummer R. Lorigan P. Steven N. Scott L. Middleton M. R. Wilson R. H. (2013). A Phase II Study of the Potent PARP Inhibitor, Rucaparib (PF-01367338, AG014699), with Temozolomide in Patients with Metastatic Melanoma Demonstrating Evidence of Chemopotentiation. Cancer Chemother. Pharmacol. 71, 1191–1199. 10.1007/s00280-013-2113-1 23423489

[B40] Spriggs D. R. Longo D. L. (2016). PARP Inhibitors in Ovarian Cancer Treatment. N. Engl. J. Med. 375, 2197–2198. 10.1056/NEJMe1612843 27959768

[B41] Tomao F. D'incalci M. Biagioli E. Peccatori F. A. Colombo N. (2017). Restoring Platinum Sensitivity in Recurrent Ovarian Cancer by Extending the Platinum-free Interval: Myth or Reality? Cancer 123, 3450–3459. 10.1002/cncr.30830 28678350

[B42] Underhill C. Toulmonde M. Bonnefoi H. (2011). A Review of PARP Inhibitors: from Bench to Bedside. Ann. Oncol. 22, 268–279. 10.1093/annonc/mdq322 20643861

[B43] Waddington T. Mambetsariev I. Pharaon R. Fricke J. Baroz A. R. Romo H. (2021). Therapeutic Potential of Olaparib in Combination with Pembrolizumab in a Young Patient with a Maternally Inherited BRCA2 Germline Variant: A Research Report. Clin. Lung Cancer 22, e703–e707. 10.1016/j.cllc.2021.01.009 33640299 PMC8978804

[B44] Yao H. Ji M. Zhu Z. Zhou J. Cao R. Chen X. (2015). Discovery of 1-substituted Benzyl-Quinazoline-2,4(1h,3h)-Dione Derivatives as Novel poly(ADP-Ribose)polymerase-1 Inhibitors. Bioorg Med. Chem. 23, 681–693. 10.1016/j.bmc.2014.12.071 25614115

[B45] Yuan Z. Chen J. Li W. Li D. Chen C. Gao C. (2017). PARP Inhibitors as Antitumor Agents: a Patent Update (2013-2015). Expert Opin. Ther. Pat. 27, 363–382. 10.1080/13543776.2017.1259413 27841036

[B46] Zhu Z. Jin J. Xue N. Song X. Chen X. (2014). Development and Validation of High-Throughput Screening Assays for poly(ADP-Ribose) Polymerase-2 Inhibitors. Anal. Biochem. 449, 188–194. 10.1016/j.ab.2013.12.028 24382396

