# Supplementary material for: A Novel PARP Inhibitor YHP-836 For the Treatment of BRCA-Deficiency Cancers
Source: Front Pharmacol. 2022 Jul 13;13:865085. doi: 10.3389/fphar.2022.865085 (PMC9326368; doi:10.3389/fphar.2022.865085)
Supplement: Supplementary file 1 [file DataSheet1.docx]

**Supplementary material**

**Scheme 1.** Reagents and conditions: a) 1-(2,2,2-trifluoroethyl)piperazine hydrochloride, HATU, HOBt, DIEA, DMF, r.t.; A mixture of compound 5-((2,4-dioxo-3,4-dihydroquinazolin-1(2*H*)-yl)methyl)-2-fluorobenzoic acid (1 equiv), HATU (2 equiv), HOBt (2 equiv), DIEA (2 equiv) and 1-(2,2,2-trifluoroethyl)piperazine hydrochloride (1.5 eqquiv) in DMF was stirred at room temperature for 20 h and then H_2_O was added to the mixture. The solution was extracted with [methylene chloride](http://www.baidu.com/link?url=9iFiFqDT-vdizI_RIDsJVbnYgzT2Dbr4079gYDQWlF_LTcNWDzaNzLm9a0Kh8sVcvQ4tGs_gQiQ_dCWjFmyLq6v3m66o1PCaqs-zLRdAXtF9GBOYUNkjNrYih4PAoqkp&wd=&eqid=89fa618d00002981000000035721a9ac) and then the organic layer was washed with brine. The combined organic layer was dried over anhydrous MgSO_4_. After filtration and concentration, the crude product was obtained and purified with column chromatography to give the target compound as white solid (40 mg, 20.2%); m.p. 128-130 °C; ^1^H-NMR (600 MHz, DMSO-*d*_6_): *δ* (ppm) 11.73 (s, 1H), 8.03 (dd, *J*_1_ = 7.8 Hz, *J*_2_ = 1.2 Hz, 1H), 7.66 (td, *J*_1_ = 7.2 Hz, *J*_2_ = 1.8 Hz, 1H), 7.41–7.44 (m, 1H), 7.35 (dd, *J*_1_ = 6.0 Hz, *J*_2_ = 1.8 Hz, 1H), 7.24–7.28 (m, 3H), 5.32 (s, 2H), 3.62 (s, 2H), 3.37 (s, 2H), 3.21 (q, *J* = 10.2 Hz, 2H), 3.15 (s, 2H), 2.66 (s, 2H); ^13^C-NMR (150 MHz, DMSO-*d*_6_): *δ* (ppm) 163.58, 161.79, 157.86 (d, *J*_CF_ = 247.2 Hz), 150.68, 140.67, 135.15, 133.14 (d, *J*_CF_ = 2.6 Hz), 129.60 (d, *J*_CF_ = 8.1 Hz), 127.63, 126.92 (d, *J*_CF_ = 3.5 Hz), 125.88 (q, *J*_CF_ = 278.4 Hz), 123.90 (d, *J*_CF_ = 18.5 Hz), 122.74, 116.16 (d, *J*_CF_ = 21.8 Hz), 116.04, 114.97, 56.46 (q, *J*_CF_ = 29.3 Hz), 52.95, 52.53, 46.47, 44.32, 41.27; HR-MS (ESI) m/z: calcd For C_22_H_21_O_3_N_4_F_4_ [M + H]^+^, 465.1544; Found, 465.1548.

Mercury-600 H-NMR YHP-836 in DMSO-*d*_6_

Mercury-600 C-NMR YHP-836 in DMSO-*d*_6_

MS

**Supplementary data**


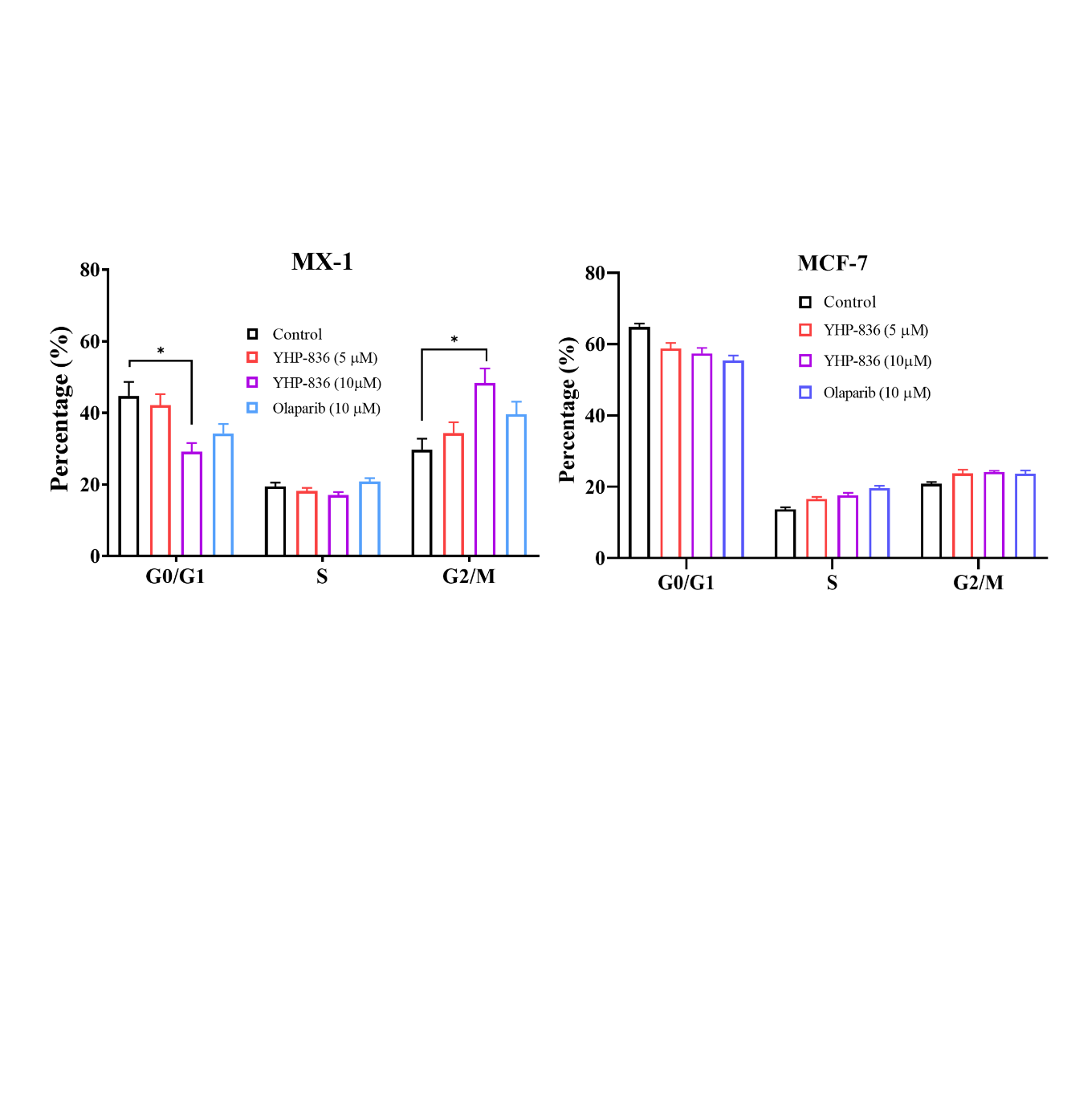


**S Fig 1**. The cell cycle analysis of MX-1 and MCF-7 cells exposure to YHP-836 or olaparib. ANOVA analysis, ^*^p<0.05 compared to control. Data are presented as mean ± SD.


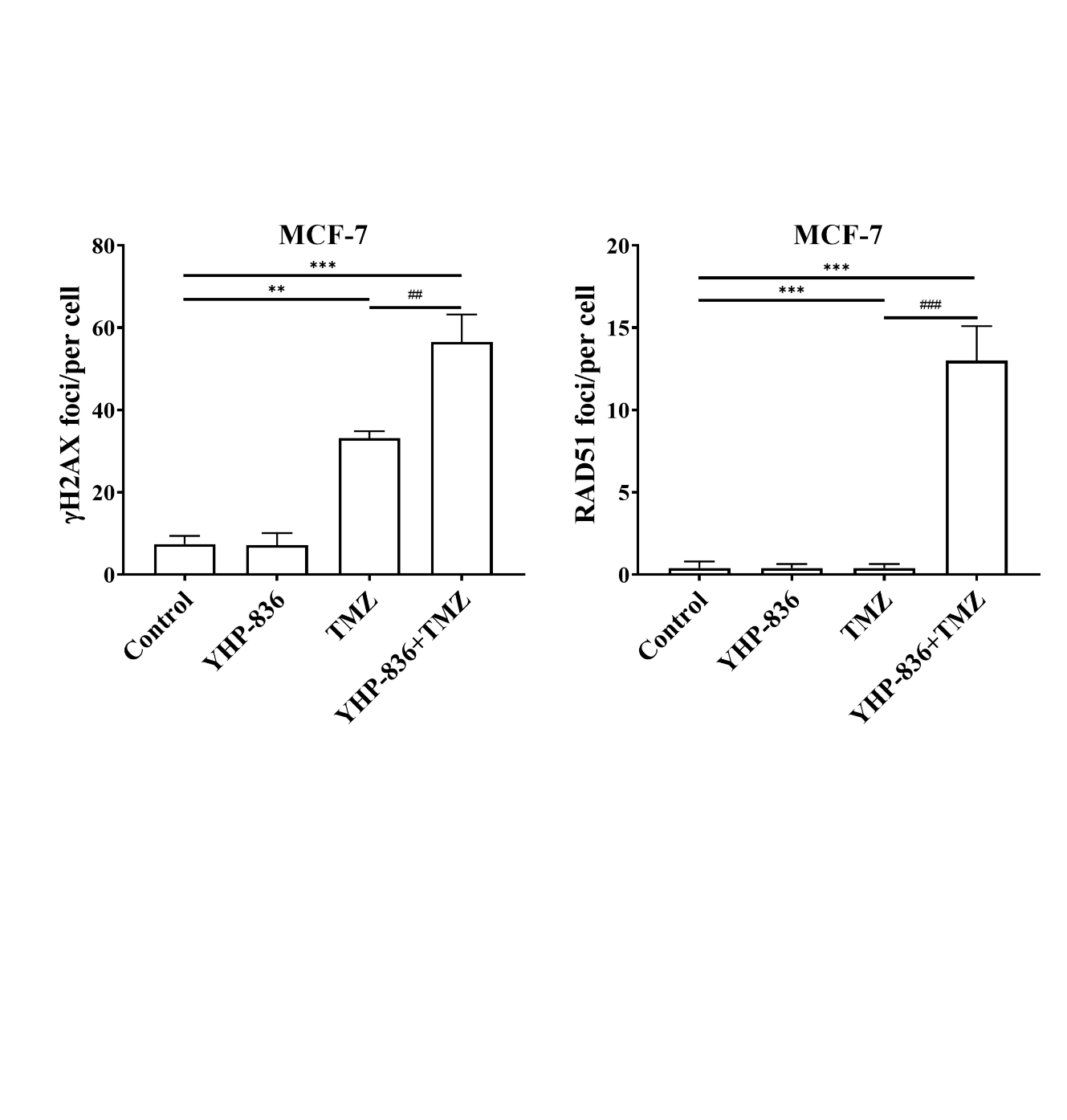


**S Fig 2**. The quantitative analysis of γ-H2AX and RAD51 foci on MCF-7 cells. ANOVA analysis, ^**^p<0.01, ^***^p<0.001 compared to control, ^##^p<0.01, ^###^p<0.001 compared to TMZ. Data are presented as mean ± SD.

**S Table 1**. Combination Index (CI) of PARP inhibitor and various chemotherapy agents in MX-1 cells

| **CI** | **YHP-836** | **Olaparib** |
| --- | --- | --- |
| TMZ | 0.188 | 0.333 |
| TPT | 0.407 | 0.637 |
| CDDP | 0.518 | 0.554 |
| ADM | 0.717 | 0.710 |

**S Table 2**. Combination Index of YHP-836 and TMZ in various cancer cells

| **CI** | **TMZ** |
| --- | --- |
| MDA-MB-436 | 0.411 |
| MDA-MB-468 | 0.252 |
| MDA-MB-453 | 0.378 |
| MCF-7 | 0.548 |
| Capan-1 | 0.407 |
| OVCAR-8 | 0.648 |
